# Cognitive performance trends among European older adults: exploring variations across cohorts, gender, and educational levels (2007–2017)

**DOI:** 10.1186/s12889-024-19123-3

**Published:** 2024-06-20

**Authors:** Johan Rehnberg, Stefan Fors, Katherine J. Ford, Anja K. Leist

**Affiliations:** 1 Aging Research Center, Karolinska Institutet and Stockholm University, Tomtebodavägen 18A, Solna, Solna, SE-171 65 Sweden; 2 Department of Public Health, Stockholm University, Albanovägen 12, Stockholm, Sweden; 3 Center for Epidemiology and Community Medicine, Region Stockholm, Stockholm, Solnavägen, 1E Sweden; 4 Department of Psychology, Carleton University, Ottawa, K1S 5B6 Canada; 5 Department of Social Sciences, University of Luxembourg, Esch-sur-Alzette, 4366 Luxembourg

**Keywords:** Delayed recall, Immediate recall, Verbal fluency, Flynn effect, Cohort improvement

## Abstract

**Background:**

This study explores recent cohort trends in cognitive performance among older Europeans from 2007 to 2017, addressing three key questions: (1) Did cognitive performance improve universally and across the performance distribution during this period? (2) Did these improvements occur across educational levels and for both men and women? (3) Can established risk factors explain these performance gains?

**Methods:**

Using data from the Survey of Health, Ageing and Retirement in Europe (SHARE) across 12 European countries, we assessed immediate recall, delayed recall, and verbal fluency in individuals aged 60 to 94 in both 2007 and 2017 (*n* = 32 773). Differences between the two time points were estimated with linear mixed effects regression models and quantile regression.

**Results:**

Cognitive performance improved in all age groups, across educational levels, and for both men and women between 2007 and 2017. Notably, improvements were more pronounced at the upper end of the performance distribution for delayed recall and verbal fluency. Education explained approximately 20% of the observed improvements. Risk factors did not explain the observed improvements.

**Conclusions:**

European cohorts of both younger-old and older adults continue to exhibit improvements in cognitive performance. Variation in the size of the cohort improvements across the performance distributions in delayed recall and in verbal fluency may contribute to growing inequalities in cognitive outcomes. Future research should further investigate the potential heterogeneity in cognitive performance gains.

**Trial registration:**

Not applicable.

**Supplementary Information:**

The online version contains supplementary material available at 10.1186/s12889-024-19123-3.

## Background

Throughout the 20th century later born cohorts have shown improvements in standardized intelligence test scores compared to earlier born cohorts, commonly referred to as the Flynn effect [1, 2]. Comparable improvements in cognitive performance have also occurred among older adults [3–7]. Generally, birth cohort increases are most pronounced in fluid-like abilities, emphasizing abstract thinking and mental speed, while smaller increases are seen in crystallized-like abilities, which involve general knowledge and vocabulary.

The question of whether secular improvements in cognitive performance will continue, or if there exist limits to cognitive plasticity, remains unanswered. In this study, we investigate several underexplored perspectives on recent cohort trends in cognitive performance among older Europeans. First, we explore cohort trends across subgroups of the population defined by sex/gender and educational levels. Second, we explore whether cohort improvements are of equal size across the range of the performance distribution overall and by educational group. We consider three indicators of cognitive performance: immediate recall, delayed recall, and verbal fluency.

Several hypotheses have been proposed to explain the improvements in cognitive performance seen in later born cohorts. Most of these theories attribute the gains to sociocultural and environmental factors, such as smaller family units [8], improved nutrition during critical pre- and post-natal periods [9], increased focus on problem-solving in the workplace [2], and the expansion and increase in quality of education. Additionally, there could be time trends in modifiable risk factors that affect cognitive outcomes, for older adults such factors include among others: smoking, excessive alcohol consumption and physical inactivity [10]. While some factors, such as smoking and education, have shown improvement in developed countries, others, like physical activity, have declined during the 20th century. The recent observations of decreasing gains in cognitive performance among later born cohorts could, therefore, be attributed to the positive effects of sociocultural, environmental, and behavioural factors having reached a saturation point for cohorts born during the second half of the 20th century. Hessel et al. [31] demonstrated that improvements in word recall among older individuals were beginning to plateau in European countries that had enjoyed higher levels of socioeconomic development for longer, compared to less developed countries. In contrast, the decline in cognitive performance observed in recent-born cohorts of older individuals in the US (born between 1948 and 1959), appears to be due to *worse* socioeconomic and demographic conditions, as well as a higher prevalence of cardiovascular risk factors and mental health problems in these cohorts [11].

Socioeconomic and environmental conditions in childhood and young adulthood are associated with cognitive outcomes later in life [12, 13]. Lack of improvement or decline in sociocultural and environmental conditions among certain population sub-groups could impact the magnitude of gains in cognitive performance observed among subsequent cohorts of older individuals. Several studies have shown that trends in cognitive performance differ across various socioeconomic and demographic groups [7, 15, 16]. For instance, Ang et al. [14] found that cohort improvements in math scores among US children aged 5 to 11 were larger in higher-educated and higher-income groups than in lower-educated and lower-income groups. However, Zheng [11] showed that cohort trends were similar in cognitive functioning across various socioeconomic position indicators in six cohorts born between 1890 and 1959 in the US. The heterogeneity in cognitive performance gains across sub-groups of the population observed in some studies highlights the need for further research to understand the development of trends and disparities in different cohorts.

Historically, men and women have had different levels of access to education, occupational opportunities, and health-related behaviours, all of which can impact cognitive outcomes [17, 18]. Among older adults, studies have shown sex differences in specific cognitive ability domains and the risk of Alzheimer’s disease [19, 20]. These differences suggest that cognitive improvements in later-born cohorts may vary by sex due to differential exposure to environmental and innate biological factors. For instance, men and women might benefit differently from advancements in education and health services, or they may be differently affected by societal changes such as increased workforce participation and shifts in gender roles. Consequently, these variations can lead to different cognitive performance gains across cohorts in older men and women.

Studies have shown that cohort gains in cognitive performance are greater among adults and younger adults with lower ability levels, compared to those with higher ability levels [21–24]. Heterogeneity in gains across the cognitive performance distribution could also be present among older persons. However, most studies that examine cohort trends in cognitive performance among older adults only estimate an average effect per cohort and do not consider distributional changes in cognitive performance that may have accompanied changes in average levels. No studies have examined whether recent cohort trends in cognitive performance among older persons are of equal size at all ability levels.

This study aims to examine cohort trends in cognitive performance between 2007 and 2017 among older adults in Europe. In addition to population cohort trends, we examine cohort trends in sub-groups of the population. We consider cohort trends in education groups, for men and women, and at different performance levels. We aim to answer the following three questions: (1) did cognitive performance increase among older adults in Europe between 2007 and 2017 universally and across the performance distribution? (2) have changes in cognitive performance between 2007 and 2017 occurred at a similar rate across educational levels and for men and women? (3) can established risk factors (smoking, Body Mass Index, depression, physical inactivity, and hearing) explain changes in cognitive performance between 2007 and 2017?

## Methods

The Survey of Health, Ageing and Retirement in Europe (SHARE) is a cross-national and longitudinal survey that collects data on health, social and economic factors among Europeans aged 50 and older [25]. Initiated in 2004, SHARE is designed to provide a comprehensive overview of the aging process in Europe by capturing a wide range of variables related to physical and mental health, socio-economic status, and social and family networks. The survey is conducted biennially and includes repeated measures from the same individuals over time as well as refreshment samples, allowing for detailed investigations of aging. Our sample includes data collected on two occasions (survey waves) 2007 and 2017 and include 12 countries that participated in both these waves (Austria, Germany, Sweden, Netherlands, Spain, Italy, France, Denmark, Greece, Switzerland, Belgium, Poland). The sample includes persons between ages 60 and 94 at both these occasions, resulting in the sample from 2007 being born between 1913 and 1947, and the sample from 2017 being born between 1923 and 1957.

### Measures of cognition

Cognitive performance was assessed with three indicators: immediate recall, delayed recall, and a verbal fluency score, with higher values indicating better cognitive performance. The memory test involved the verbal registration and recall of a list of 10 words. Respondents hear the complete list once, and the test is administered twice: immediately after the encoding phase (immediate recall) and again at the end of the cognitive function module (delayed recall). The raw total scores for both tests correspond to the number of words the respondent recalled. The verbal fluency test measures the number of animals that the respondent can name within one minute. The test format used by SHARE is derived from the Telephone Interview for Cognitive Status-Modified (TICS-M), a cognitive assessment that can be administered both in person and by telephone [26]. Distributions of the cognitive variables are shown in Supplementary Fig. 1. Respondents with missing data on any of the cognitive variables were excluded. For descriptive purposes (Table 1), raw scores are presented. In the regression models, standardized variables were used, where each individual’s score was subtracted by the mean and then divided by the standard deviation, resulting in variables with mean 0 and a standard deviation of 1.

### Other variables

Adapting the three-level educational categorisation that Eurostat uses in official statistics we coded the original ISCED 1997 educational categories into low (ISCED 0–2), medium (ISCED 3–4), and high (ISCED 5–6). Additionally, analyses were either adjusted for or stratified by sex/gender and age.

### Risk factors

To examine whether risk factors could explain changes in cognitive gains, we selected five risk factors that were available in the SHARE dataset and that have previously been identified as modifiable and affecting cognitive outcomes (dementia) in later life [10]. These were: smoking, Body Mass Index (BMI), depression, physical inactivity, and hearing. Smoking was measured as a binary variable coded as 1 if the respondent had ever smoked daily. BMI was measured as a continuous variable. Depression was measured using the EURO-D depression scale that ranged from 0 to 12, where higher values indicate more depressive symptoms. Physical inactivity was measured by a binary indicator where 1 correspond to the respondent never participating in vigorous or moderate physical activity. Hearing was measured on a 5-point scale where 1 indicated excellent hearing and 5 indicated poor hearing. Each risk factor was measured during the same survey wave as the cognitive measures. To minimise potential bias from item-nonresponse, these variables were obtained from the multiple imputed datasets provided by SHARE. For 131 persons, imputation was not valid, and as a result, these individuals were excluded from the analyses that included the risk factors (model 3).

### Analytical strategy

To analyse cohort trends between the two included time points (2007 and 2017), we fit linear mixed effects regression models for each of the three standardized cognitive performance outcomes. The main exposure was a binary indicator that identified the 2007 and 2017 cohorts. Mixed effect regression methods are suitable when observations in the data are clustered and therefore violate the assumption that observations must be independent from each other. In this data, observations were clustered within countries, and subsequently, country was specified as the grouping factor to allow for random intercepts within each country and a dichotomous variable indicating year of measurement (2007 or 2017) was included as a random effect, allowing for random slopes within each country. The fixed effects part in *model 1* included: sex, age, age squared, measurement year, and three interaction terms between: age and measurement year; age and sex; sex and measurement year. The measurement year variable estimates change in the cognitive performance outcomes and the interaction terms allowed for different estimates across ages and by sex. *Model 2* included all variables from model 1 with the addition of education and two interaction terms between education and age, and education and measurement year. *Model 3* included all variables from model 2, with the addition of the five risk factors that may influence cognitive performance. Marginal effects were estimated in order to ease the interpretation of the average effect across all co-variates and interaction terms.

To analyse cohort trends at different parts of the performance distributions between 2007 and 2017 we used conditional quantile regression (CQR). CQR is appropriate for exploring whether the effect of interest is uniform or varies across the conditional outcome distribution [27, 28]. Like the linear mixed effects models, a measurement year variable (wave) was included in the model that quantified the difference in the cognitive outcome between 2007 and 2017. To keep the models parsimonious, we only included the variables: measurement year, sex, and age. The coefficients estimated with the CQR are interpreted as the difference in the cognitive outcome between the two years at each decile of the cognitive outcome distributions. The quantile regression estimates were compared against the estimates obtained from a linear regression model with the same model specification.

In all analyses we used calibrated cross-sectional weights provided in the SHARE data for respondents participating in the two relevant waves. These weights adjust for the sampling procedures in each country and incorporates a calibration approach aimed at aligning the sample and population distributions of benchmark variables. This helps mitigate selection bias resulting from nonresponse errors (see the SHARE release guide for additional information [29]).

### Sensitivity analyses

Age was modelled including a polynomial term in the main analyses to accommodate a simple curvilinear shape in the linear effect models. To test whether a more flexible model would yield different shapes age we tested several model specifications that included age estimated with b-splines and with different knot placements, these models showed similar curvilinear shapes to the basic polynomial term (results not shown). To examine whether the results varied between countries, we performed country-specific analyses for model 1 (see Supplementary Figs. 2–4). To examine the potential impact of practice (or retest) effects, we performed analyses with an additional variable that measured the number of previous surveys that they respondents had participated in [30] (see Supplementary Tables 1–2).

## Results

Table 1 shows descriptive statistics for the two cohorts in 2007 and 2017 by younger-old (60–74) and older-old (75+) age groups. There was a substantial increase in education over the two time points. Among those aged 60–74, the percentage of individuals with high education increased from 32% in 2007 to 39% in 2017, and for those aged 75+, the corresponding increase was from 20.9 to 26.1%. Both age groups experienced improvements in all three cognitive outcomes between 2007 and 2017. The risk factors BMI, depressive symptoms, and hearing remained stable over this period. The prevalence of persons that ever smoked increased somewhat and the prevalence of physical inactivity decreased somewhat.

**Table 1:** Descriptive statistics by age (60–74 and 75+) and between the two measurement years (2007 and 2017) (*n* = 32 773)

	Survey wave 2007	Survey wave 2017
Age, mean (sd)	70.8 (7.8)	72.8 (7.9)
Sex n, (% women)		
60–74	7180 (53.0%)	4548 (56.9%)
75+	3508 (56.8%)	2841 (56.2%)
Education, n (%)		
60–74		
Low	6856 (50.6%)	2993 (37.5%)
Medium	2323 (17.1%)	1910 (23.9%)
High	4374 (32.3%)	3088 (38.6%)
75+		
Low	4276 (69.2%)	2921 (57.8%)
Medium	611 (9.9%)	816 (16.1%)
High	1288 (20.9%)	1317 (26.1%)
Immediate recall, mean (sd)		
60–74	5.0 (1.7)	5.5 (1.6)
75+	3.7 (1.8)	4.3 (1.8)
Delayed recall, mean (sd)		
60–74	3.5 (1.9)	4.2 (2.0)
75+	2.3 (1.8)	2.8 (2.0)
Verbal Fluency, mean (sd)		
60–74	18.8 (7.1)	20.3 (7.5)
75+	14.7 (6.7)	16.1 (7.4)
BMI, mean (sd)		
60–74	27.0 (4.3)	27.1 (4.5)
75+	26.2 (4.2)	26.6 (4.3)
Depressive symptoms, mean (sd)		
60–74	2.2 (2.2)	2.1 (2.1)
75+	2.9 (2.5)	2.9 (2.4)
Hearing, 1- excellent, 5-poor, mean (sd)		
60–74	2.6 (1.01)	2.6 (0.96)
75+	3.1 (1.03)	3.0 (0.99)
Ever smoked, n (%)		
60–74	6158 (45.6%)	4032 (50.5%)
75+	2111 (34.8%)	1899 (37.6%)
Physical inactivity, n (%)		
60–74	1173 (8.7%)	548 (6.9%)
75+	1581 (26.0%)	1132 (22.4%)

*Note* all variables are presented as unweighted raw (non-standardized) scores; sd, standard deviation; n, number of observations

Supplementary Fig. 5a-c shows standardized scores for immediate recall, delayed recall, and verbal fluency from age 60 to age 94 in 2007 and in 2017, estimated from a linear mixed effects model adjusted for age and sex (see Supplementary Table 3, model 1 for the full models). The scores increased between 2007 and 2017 in all three outcomes and across all ages. The average increase was 0.342 standard deviations for immediate recall, 0.340 for delayed recall, and 0.276 for verbal fluency (see Table 2, model 1). The increase between the two measurement periods was slightly smaller in older ages.

**Fig. 1:**
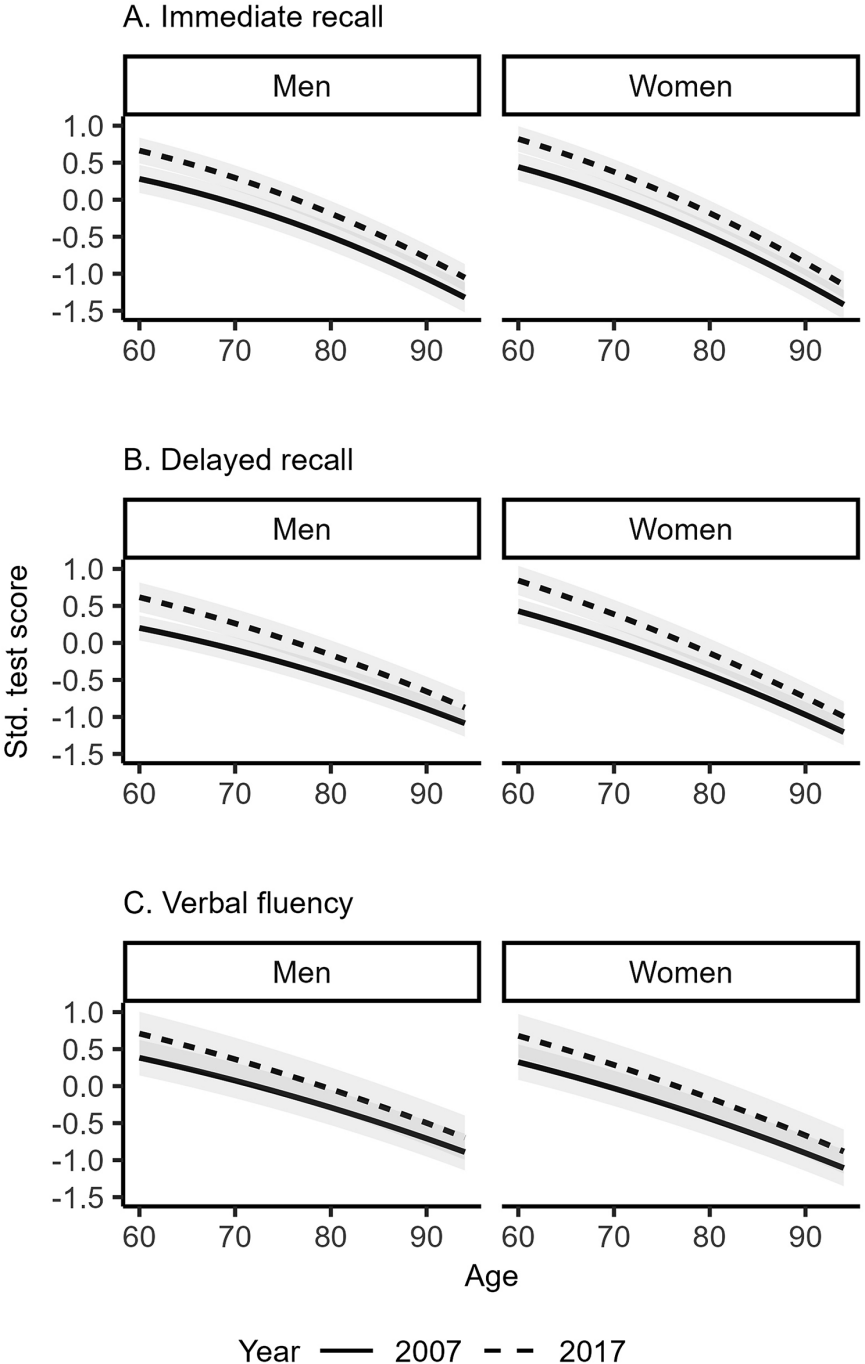
**a**–**c** Immediate recall (**a**), delayed recall (**b**), and verbal fluency (**c**) scores by age and sex in 2007 and 2017, *n* = 32 773 (model 1). Grey bands indicate 95% confidence intervals. The y-axis values indicate standardized test scores for each cognitive outcome

Figure 1a-c shows the standardized scores for immediate recall scores, delayed recall scores, and verbal fluency scores from age 60 to age 94 in 2007 and in 2017 estimated separately by sex from a linear mixed effects model adjusted for age and sex (see Supplementary Table 3, model 1). Women scored slightly higher than men on immediate and on delayed recall in the younger age categories, but not in the older ages. The increase in cognitive performance scores were similar for both men and women with no discernible differences.

**Fig. 2:**
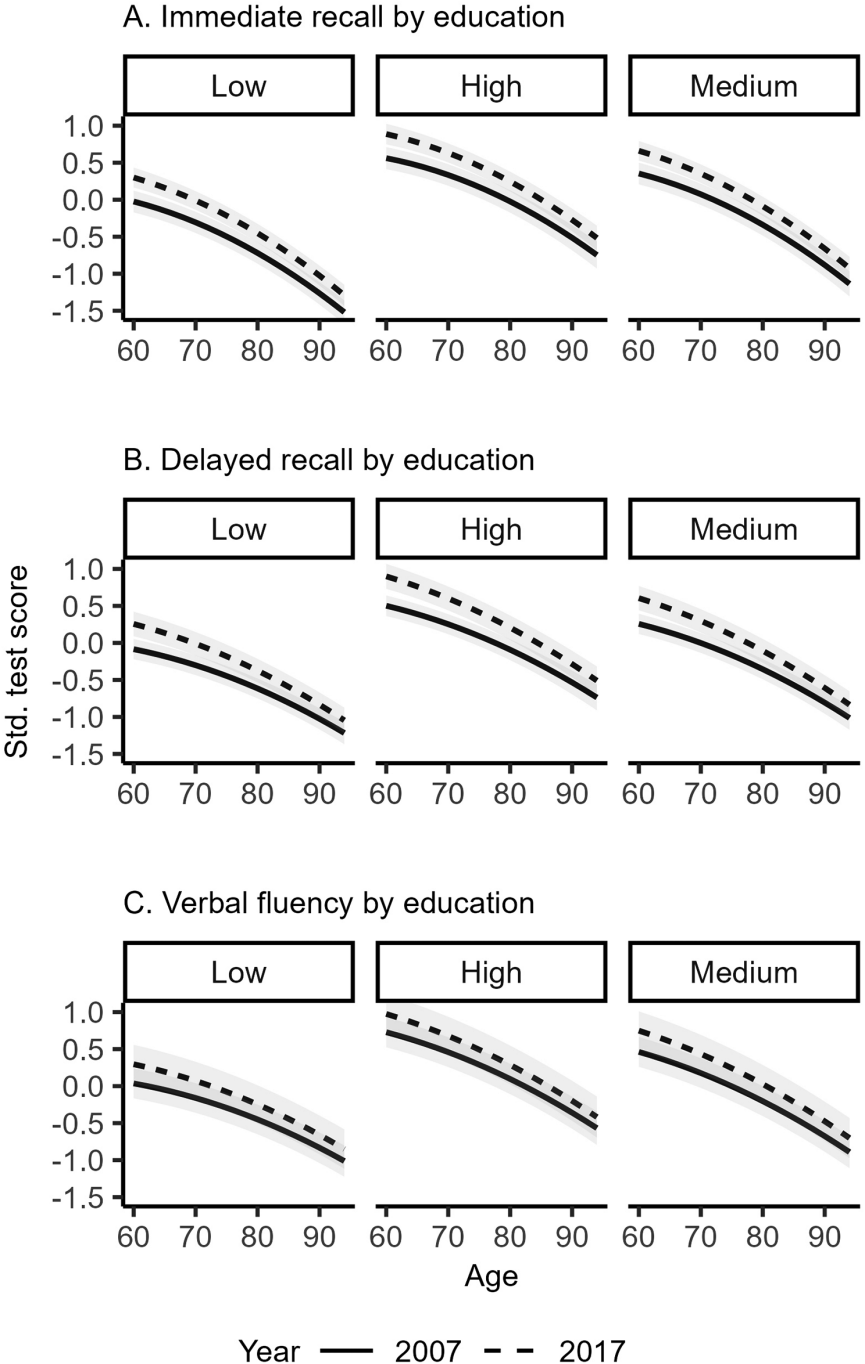
**a**–**c** Immediate recall (**a**), delayed recall (**b**), and verbal fluency (**c**) scores by age and education in 2007 and 2017, *n* = 32 773 (model 2). Grey bands indicate 95% confidence intervals. The y-axis values indicate standardized test scores for each cognitive outcome

Figure 2a-c shows the standardized scores for immediate recall, delayed recall, and verbal fluency from age 60 to age 94 in 2007 and in 2017 estimated separately by education from a linear mixed effects model adjusted for age, sex, and education (see Supplementary Table 3, model 2). Persons with higher education scored higher on all three cognitive outcomes compared to lower educated persons and the increase in cognitive performance scores were similar across all educational groups.

**Fig. 3:**
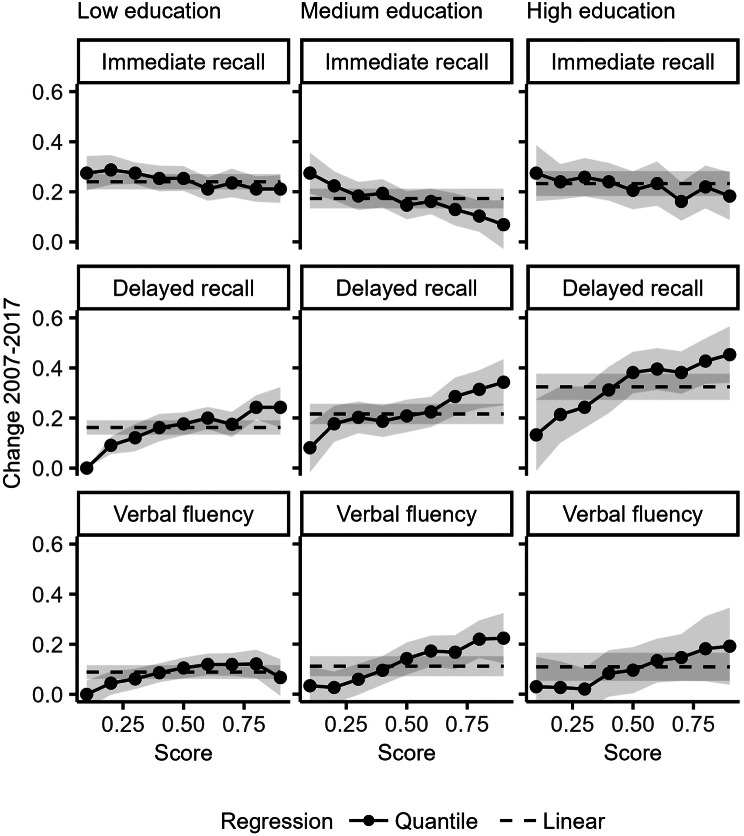
Change in delayed recall, immediate recall, and verbal fluency between 2007 and 2017 stratified by education. Dotted line shows the average change in the entire sample estimated from a linear regression, full solid lines show changes at every 10th percentile estimated from quantile regression. Grey bands indicate 95% Confidence intervals. Adjusted for age and sex (*n* = 32 773). The y-axis values indicate standardized test scores for each cognitive outcome

Supplementary Fig. 6 shows the increase in the three cognitive outcomes between 2007 and 2017 at different quantiles of the outcome distributions and Fig. 3 shows the same results stratified by educational level. The results indicate some variability in cohort gains at different levels of the outcome distributions. In delayed recall, the changes in cognitive performance between 2007 and 2017 were less pronounced at the lower end of the score distributions but more substantial at the upper end for all three educational groups. Similarly, in verbal fluency, we observed a similar pattern of larger gains at the upper end of the score distribution among persons with medium and high education. The results suggest an increase in heterogeneity in delayed recall and in verbal fluency between 2007 and 2017, this result is further supported by larger standard deviations at the later measurement point in delayed recall and verbal fluency (see supplementary Table 5).

To examine whether modifiable risk factors contribute to gains in the three cognitive outcomes between 2007 and 2017 we adjust the linear mixed effects model for five risk factors. The results presented in Table 2 show three different models: model 1 is adjusted for measurement year, age, and sex, model 2 is additionally adjusted for education, and model 3 is additionally adjusted for smoking, BMI, depression, physical inactivity, and hearing. The average marginal effect was estimated from the measurement year variable and summarizes the average increase between 2007 and 2017 across the marginal distribution of the three cognitive outcomes. The cohort gain was attenuated when adjusting for education (model 1 vs. model 2), although the confidence intervals overlapped the point estimates between the models for delayed recall and verbal fluency. The five risk factors explained only a minor part of the cohort gains in the three cognitive outcomes between 2007 and 2017 (model 2 vs. model 3, Table 2). For example, in immediate recall, the coefficient 0.267 from model 2 (Table 2) indicate that the average increase in immediate recall was 0.267 standard deviations between the 2007 cohort and the 2017 cohort. After adjusting the immediate recall model 2 for the five risk factors, the cohort gains remain at a similar level at 0.253 standard deviations (see Table 2, model 3).

**Table 2:** The average cohort gain in immediate recall, delayed recall, and verbal fluency standardized score. Estimated from a linear mixed effects model. (*n* = 32 773 in model 1–2; *n* = 32 642 in model 3)

		Average gain between 2007–2017	LCI	UCI
Immediate recall				
	Model 1	0.342	0.284	0.401
	Model 2	0.267	0.205	0.328
	Model 3	0.253	0.189	0.317
Delayed recall				
	Model 1	0.340	0.254	0.426
	Model 2	0.276	0.189	0.364
	Model 3	0.265	0.177	0.354
Verbal fluency				
	Model 1	0.276	0.164	0.388
	Model 2	0.209	0.087	0.331
	Model 3	0.193	0.075	0.311

*Note* LCI, lower confidence interval; UCI, upper confidence interval; n, number of observations. Model 1 adjusted for age, sex, and measurement year; model 2 adjusted for model 1 + education; model 3 adjusted for model 2 + smoking, BMI, depression, physical inactivity, and hearing

## Discussion

In this study, we investigated trends in delayed recall, immediate recall, and verbal fluency among older adults aged 60 to 94 between 2007 and 2017. We examined trends in the entire population, in educational groups, among men and women, and at different levels of the performance distribution. Despite observations of decelerated [31] or even negative cohort gains [11], our analysis revealed gains in all three cognitive outcomes in all ages, all educational groups, among both men and women and across the entire cognitive distributions. In delayed recall and to a lesser extent in verbal fluency, gains between 2007 and 2017 were larger at the top of the score distribution and smaller at the bottom. Education, but not risk factors that influence cognitive outcomes, explained some of the gains the three outcomes.

Education accounted for approximately 20% of the overall improvement observed in the three cognitive outcomes between 2007 and 2017. This finding aligns with cognitive development theories that propose the protective influence of education and/or ability on cognitive function, such as the cognitive reserve concept [32, 33] or the scaffolding theory [34]. This effect may stem from an increased impact of education, possibly linked to improved educational quality, changes in the composition of the study population concerning education, or a combination of both factors. Notably, the improvements observed in individuals at the highest performance levels may suggest that this socioeconomic group selectively seeks and benefits from opportunities for lifelong learning or engages in specific activities like computer use [35] that may contribute to cognitive gains.

Several previous studies has demonstrated larger cohort gains in cognition at lower performance levels among younger adults and adults [21–24]. However, the examination of cohort gains across the cognitive performance distributions among older individuals is scarce in the literature. Interestingly, our results revealed opposite patterns among older adults: cohort gains were smaller at the bottom of the score distributions and larger at the top of the score distributions in delayed recall and verbal fluency. The fact that individuals at the top of the performance distribution showed strongest secular improvements suggests that opportunities for cognitive development [36] are not equally distributed in the population, and existing inequalities may have been aggravating during the window of observation. These findings highlight the importance of studying heterogeneity in cognitive gains across the outcome distribution and future studies should explore these patterns further. Additionally, the observed cognitive gains across the entire distribution in delayed recall and verbal fluency suggest an absence of clear ceiling effects that would otherwise mask improvements among high performers in these cognitive outcomes.

Cohort gains could arise from various factors, including societal conditions or shifts in individual socioeconomic and health risk burdens. We tested whether five established and modifiable risk factors for cognitive outcomes among older adults could explain the cohort gains that we observed. However, these risk factors did not explain any substantial part of the cohort gains between 2007 and 2017. Several reasons might have caused this. First, the prevalence of these risk factors remained similar during both periods, indicating no substantial shift in exposure between the cohorts. Second, our measurement only captures these risk factors at a single point in time, without considering life-long exposure. Third, reverse causation may play a role, where better cognitive health leads to improvements in these risk factors.

The present study has several limitations. The data from the Survey of Health, Ageing and Retirement in Europe is one of the largest datasets on older adults in Europe and covers a diverse range of older adults. However, one drawback is that the sample sizes are limited when breaking them down by country and we can therefore not make definite claims for cohort trends in specific countries. Country stratified sensitivity analyses (Supplementary Figs. 2–4) show cohort gains in the three cognitive outcomes by country. While the levels and the size of cohort gains varied between countries, all showed similar age patterns and clear cohort gains except for verbal fluency in Greece. The similar result across all countries is reassuring, however, we refrain from interpreting results from specific countries due to the limited country sample sizes.

The challenges associated with disentangling age, period, and cohort effects have been extensively discussed previously [37]. While the epidemiological definition of cohort effects (age by period interaction) is depicted in Figs. 1 and 2 and Supplementary Fig. 5, the estimation of the average increase in cognitive performance across all ages included in the study between 2007 and 2017 in Table 2 resembles a period effect. However, we interpret this period effect as a direct consequence of the cohorts present during these two periods, similar to approaches in previous studies examining the Flynn effect [2].

Practice effects leading to improvements in cognitive test scores for repeated assessments are well-documented, yet there is no definitive solution for adjusting these effects. One approach that we adopted was to adjust for the respondents’ participation in previous waves (see e.g [30]). This adjustment resulted in a slight attenuation of cohort gains in immediate recall and delayed recall, while the attenuation was somewhat more pronounced for cohort gains in verbal fluency. Specifically, the average cohort gain in verbal fluency decreased from 0.276 standard deviations (Table 2, model 1) to 0.225 standard deviations (Supplementary Table 1, model 1). However, this method of modelling practice effects is inherently correlated with age (time), as individuals who have participated in more waves have also aged. Nevertheless, even with these adjustments in place, cohort gains remained substantial.

Healthy survivor bias may influence cognitive outcomes, for example the cognitive functioning of later-born cohorts may have been less influenced by healthy survivor bias, given the decrease in death rates between 2007 and 2017. However, since a larger number of individuals with potentially poorer health survived in the later-born cohorts assessed in 2017, this bias could weaken gains in cognitive performance. If the survival rates had remained consistent across both years, the observed cohort gains in cognitive functioning might have been even more pronounced.

We aimed to examine cohort trends in cognitive performance across different ages rather than focusing on longitudinal changes within the same individuals. Therefore, while some participants are present in both waves, they are treated as separate observations at each time point. This approach allows us to compare cognitive performance at the same ages across two different time points, ensuring that individuals are not compared to their own previous measures. Additionally, the sensitivity test for practice effects indicates that repeated participation by respondents did not significantly affect the results.

## Conclusions

The analytical approach in this study, focusing on two cohorts, limits our ability to interpret the deceleration of cohort gains over longer time periods, as previously observed [31]. Instead, our findings revealed that recent European cohorts of both younger-old and older adults continue to demonstrate improvements in cognitive performance. Notably, these improvements were consistent across educational levels and for both men and women. Additionally, we noted variations in the magnitude of improvements across performance distributions, with more pronounced improvements observed at the higher end of the distribution in delayed recall and verbal fluency. These trends could potentially contribute to widening inequalities in cognitive outcomes. Future research should further investigate the potential heterogeneity in cognitive performance gain.

The Authors declares that there is no conflict of interest.

### Electronic supplementary material

Below is the link to the electronic supplementary material.

Supplementary Material 1

## Data Availability

The data used in this study was obtained from the Survey of Health, Ageing and Retirement in Europe (SHARE). Access to the SHARE data is provided free of charge for scientific use, see the SHARE website www.share-eric.eu.
