# Supplementary material for: Cognitive performance trends among European older adults: exploring variations across cohorts, gender, and educational levels (2007–2017)
Source: BMC Public Health. 2024 Jun 20;24:1646. doi: 10.1186/s12889-024-19123-3 (PMC11188163; doi:10.1186/s12889-024-19123-3)
Supplement: Supplementary file 1 — Supplementary Material 1 [file 12889_2024_19123_MOESM1_ESM.pdf]

Supplementary material: Supplementary data

Title: Cognitive performance trends among European older adults: exploring variations across cohorts, gender, and educational levels (2007-2017)

Content

Supplementary table 1-5

Supplementary figure 1-6

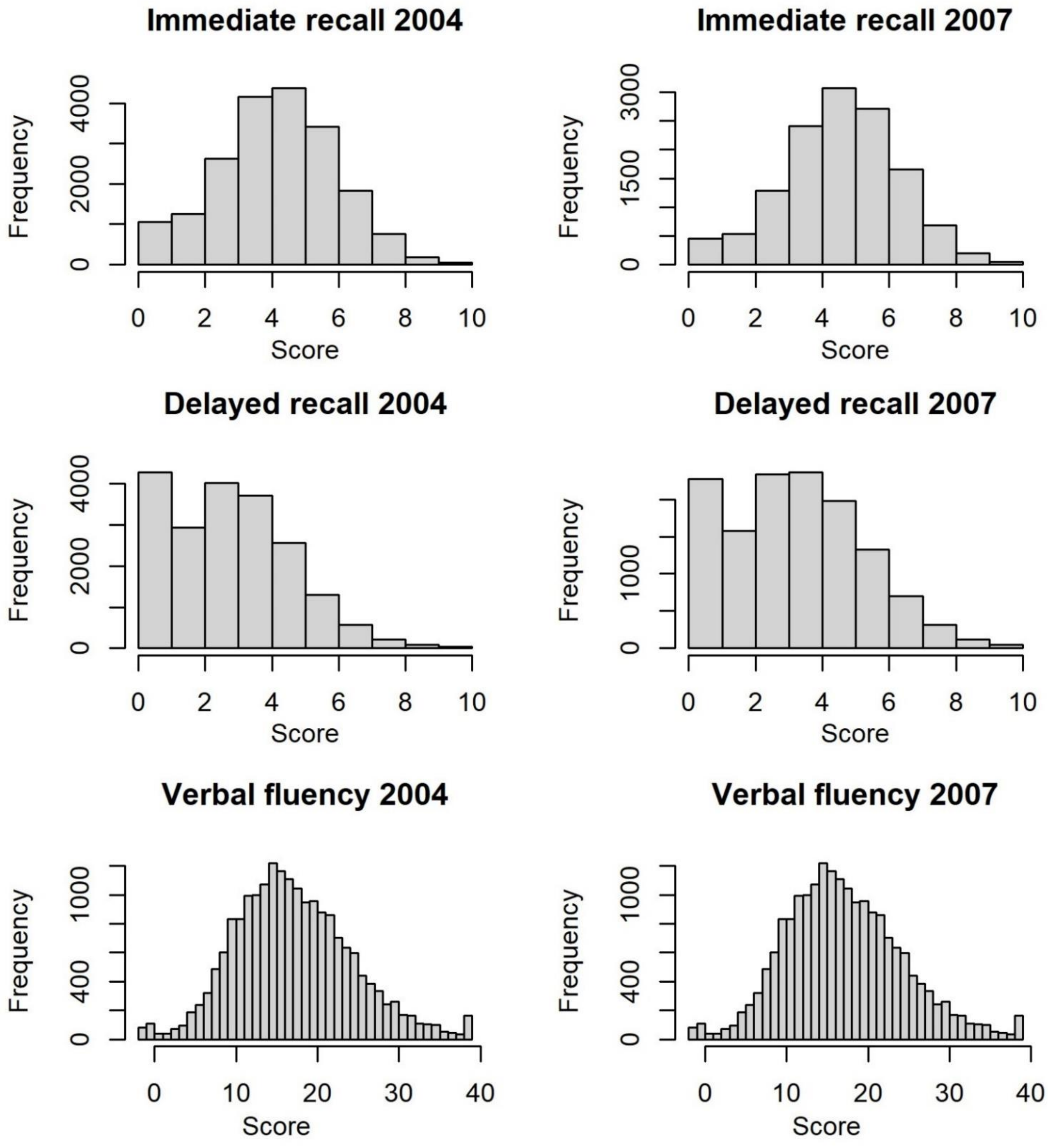

Supplementary Figure 1. Distributions for the cognitive measures: Immediate recall, delayed recall, and verbal fluency and 2017.

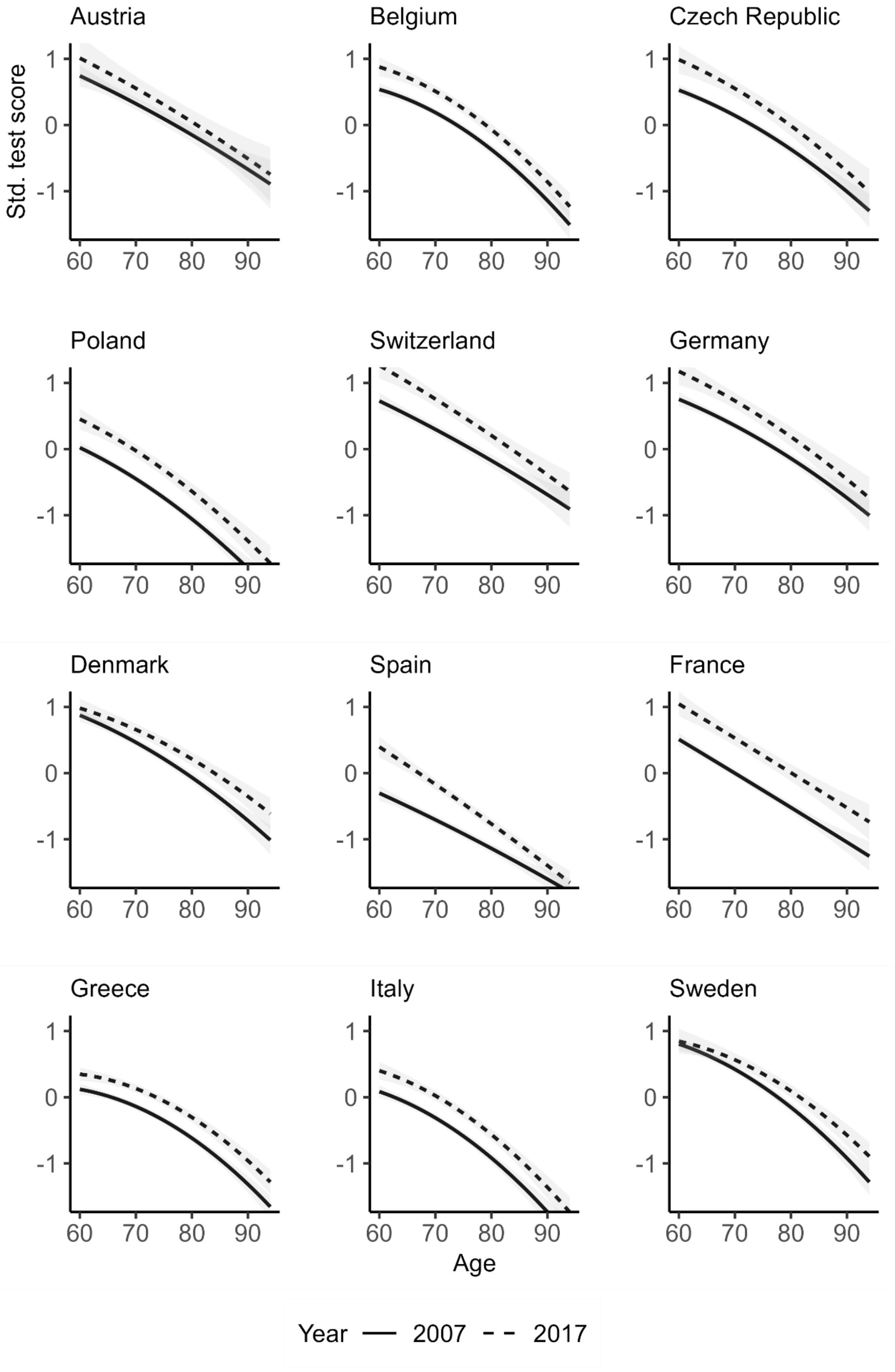

Supplementary Figure 2. Immediate recall scores by country in 2007 and 2017.

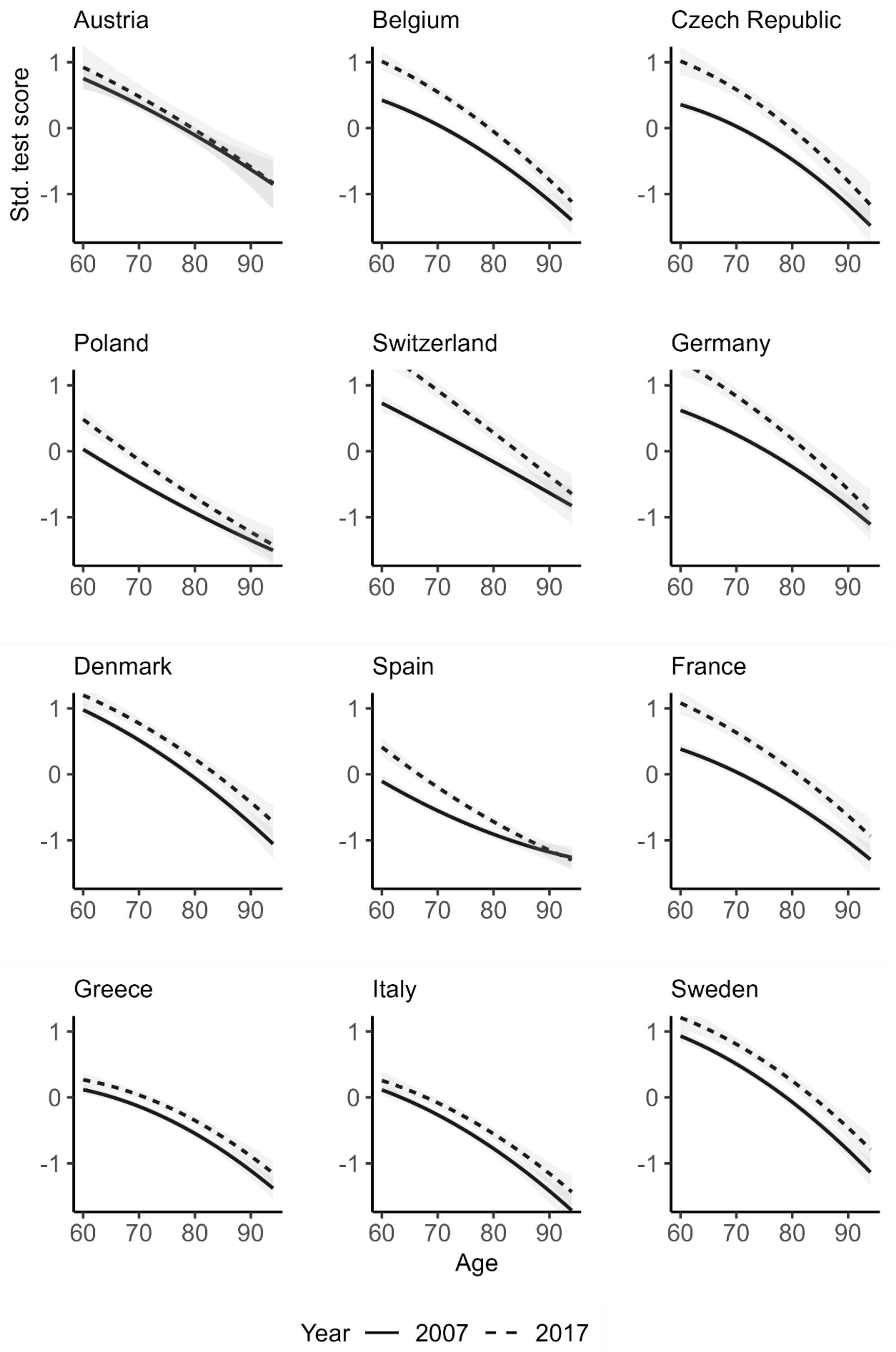

Supplementary Figure 3. Delayed recall scores by country in 2007 and 2017.

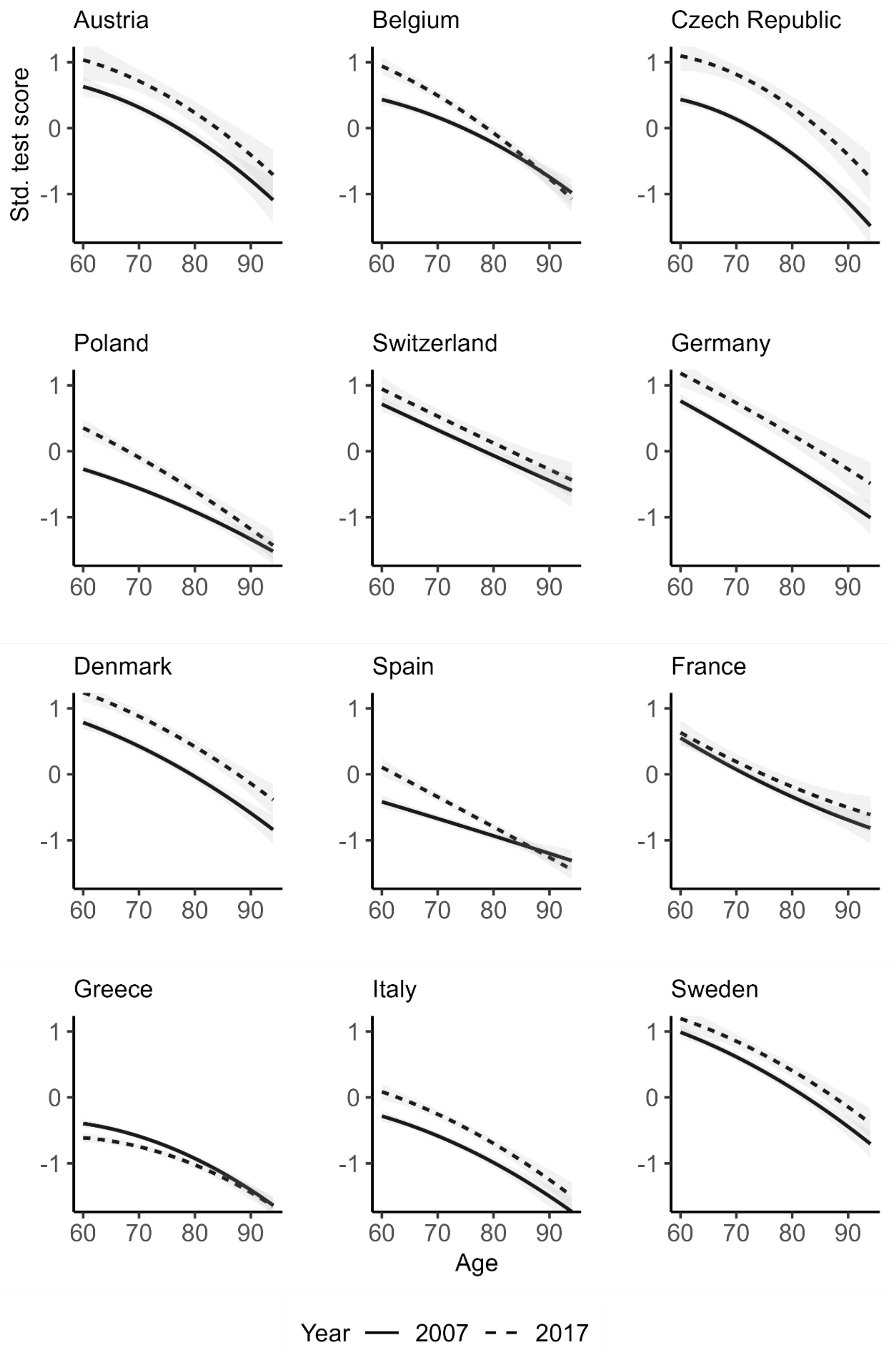

Supplementary Figure 4. Verbal fluency scores by country in 2007 and 2017.

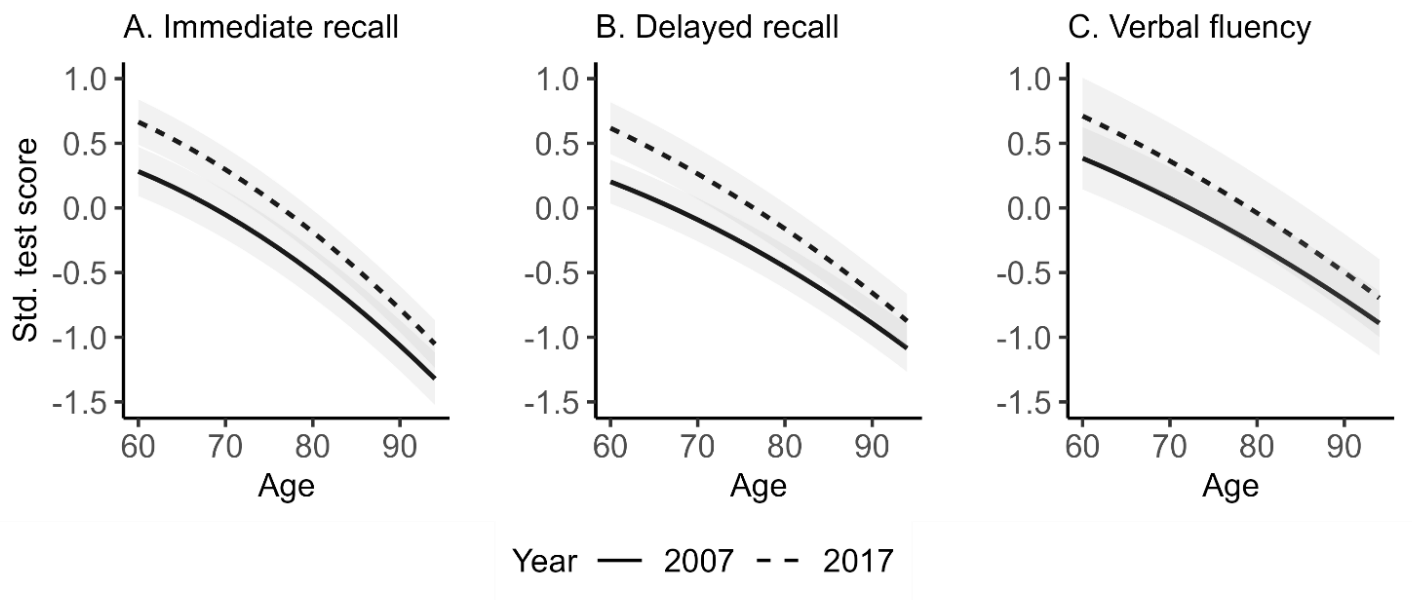

Supplementary Figure 5a-c. Immediate recall (a), delayed recall (b), and verbal fluency (c) scores by age in 2007 and 2017, n=32 773 (model 1). Grey bands indicate 95 % confidence intervals. See supplementary table 3 for full models.

Supplementary Table 1. The average cohort gain in immediate recall, delayed recall, and verbal fluency score adjusted for number of previous surveys that the respondent participated in (re-test/practice effect). Estimated from linear mixed effects regressions, model 1-3 are the same as in Supplementary Table 3 with the addition of a variable that indicates *number of previous surveys that the respondent participated in*.

		Average gain between 2007-2017	LCI, 95%	UCI, 95%
Immediate recall				
	Model 1	0.316	0.257	0.375
	Model 2	0.245	0.184	0.306
	Model 3	0.237	0.173	0.301
Delayed recall				
	Model 1	0.317	0.235	0.400
	Model 2	0.258	0.173	0.342
	Model 3	0.251	0.164	0.337
Verbal fluency				
	Model 1	0.225	0.121	0.328
	Model 2	0.162	0.049	0.276
	Model 3	0.152	0.041	0.262

Note: Model 1 adjusted for number of previous surveys, age, sex, and measurement year; model 2 adjusted for model 1 + education; model 3 adjusted for model 2 + smoking, BMI, depression, physical inactivity, and hearing. LCI, lower confidence interval; UCI, upper confidence interval.

Supplementary table 2. Coefficient for the variable *number of previous surveys that the respondent participated in* (ranging from 1-5) for models estimating: immediate recall, delayed recall, and verbal fluency score. Estimated from linear mixed effects model, see Supplementary Table 1.

		Coefficient	LCI, 95%	UCI, 95%
Immediate recall				
	Model 1	0.031	0.023	0.040
	Model 2	0.026	0.018	0.035
	Model 3	0.019	0.011	0.027
Delayed recall				
	Model 1	0.027	0.019	0.036
	Model 2	0.023	0.015	0.031
	Model 3	0.018	0.010	0.026
Verbal fluency				
	Model 1	0.063	0.054	0.071
	Model 2	0.058	0.049	0.066
	Model 3	0.051	0.043	0.059

Note: Model 1 adjusted for number of previous surveys, age, sex, and measurement year; model 2 adjusted for model 1 + education; model 3 adjusted for model 2 + smoking, BMI, depression, physical inactivity, and hearing. LCI, lower confidence interval; UCI, upper confidence interval.

Supplementary table 3. Linear mixed effect models for outcomes: immediate recall, delayed recall, and verbal fluency. Figure 1, Figure 2, Supplementary Figure 5, and Table 2 are estimated based on these models.

	**Model 1** Fig. 1, Suppl. Fig. 1., Table 2 n=32 773			**Model 2** Fig. 2, table 2 n=32 773			**Model 3** Table 2 n=32 642		
**Immediate recall**									
Random effects	*Estimate*	*Std.Dev.*		*Estimate*	*Std.Dev.*	*P-value*	*Estimate*	*Std.Dev.*	
Country (random intercept)	0.108	0.329		0.062	0.250		0.039	0.198	
Wave	0.008	0.089		0.009	0.096		0.010	0.100	
Fixed effects	*Estimate*	*Std.Error*	*P-value*	*Estimate*	*Std.Error*	*P-value*	*Estimate*	*Std. Error*	*P-value*
(Intercept)	-0.092	0.399	0.817	-1.093	0.402	0.007	-0.325	0.393	0.409
Wave(1=2017)	0.584	0.107	0.000	0.493	0.112	0.000	0.448	0.110	0.000
Age	0.040	0.011	0.000	0.057	0.011	0.000	0.038	0.011	0.000
Sex(women=1)	0.613	0.090	0.000	0.536	0.089	0.000	0.514	0.086	0.000
Age squared	-0.001	0.000	0.000	-0.001	0.000	0.000	0.000	0.000	0.000
High education (ref. low)				0.257	0.138	0.064	0.319	0.135	0.018
Medium education (ref. low)				0.368	0.103	0.000	0.434	0.101	0.000
Ever smoked							0.050	0.010	0.000
BMI							0.000	0.001	0.710
Depression (eurod)							-0.061	0.002	0.000
Physical inactivity							-0.252	0.014	0.000
Hearing (1-excellent, 5-poor)							-0.071	0.005	0.000
Wave:age	-0.003	0.001	0.017	-0.003	0.001	0.047	-0.002	0.001	0.073
Wave:sex	-0.004	0.022	0.859	-0.034	0.022	0.123	-0.030	0.021	0.160
Age:sex	-0.008	0.001	0.000	-0.005	0.001	0.000	-0.004	0.001	0.001
Age:high educ.				0.005	0.002	0.005	0.004	0.002	0.061
Age:medium educ				0.000	0.001	0.912	-0.001	0.001	0.300
Wave:high educ.				0.002	0.035	0.964	0.006	0.034	0.856
Wave:medium educ.				-0.017	0.028	0.527	-0.008	0.027	0.753
**Delayed recall**									
Random effects	*Estimate*	*Std.Dev.*		*Estimate*	*Std.Error*		*Estimate*	*Std.Dev.*	
Country (random intercept)	0.085	0.291		0.054	0.232		0.035	0.186	
Wave	0.020	0.142		0.021	0.146		0.022	0.147	
Fixed effects	*Estimate*	*Std.Error*	*P-value*	*Estimate*	*Std.Dev.*	*P-value*	*Estimate*	*Std. Error*	*P-value*
(Intercept)	0.483	0.389	0.215	-0.809	0.396	0.041	-0.183	0.391	0.640
Wave(1=2017)	0.774	0.110	0.000	0.639	0.115	0.000	0.627	0.114	0.000
Age	0.017	0.010	0.114	0.041	0.011	0.000	0.026	0.010	0.013
Sex(women=1)	0.840	0.089	0.000	0.822	0.087	0.000	0.817	0.086	0.000
Age squared	0.000	0.000	0.000	0.000	0.000	0.000	0.000	0.000	0.000
High education (ref. low)				0.775	0.136	0.000	0.811	0.134	0.000
Medium education (ref. low)				0.585	0.102	0.000	0.592	0.100	0.000
Ever smoked							0.044	0.010	0.000
BMI							-0.001	0.001	0.603

Depression (eurod)							-0.057	0.002	0.000
Physical inactivity							-0.121	0.013	0.000
Hearing (1-excellent, 5-poor)							-0.059	0.005	0.000
Wave:age	-0.006	0.001	0.000	-0.005	0.001	0.000	-0.005	0.001	0.000
Wave:sex	-0.001	0.022	0.964	-0.022	0.022	0.309	-0.021	0.021	0.330
Age:sex	-0.010	0.001	0.000	-0.009	0.001	0.000	-0.008	0.001	0.000
Age:high educ.				-0.003	0.002	0.105	-0.005	0.002	0.019
Age:medium educ				-0.004	0.001	0.005	-0.005	0.001	0.001
Wave:high educ.				0.057	0.035	0.098	0.061	0.034	0.074
Wave:medium educ.				0.008	0.028	0.779	0.012	0.027	0.667

Verbal fluency

Random effects	*Estimate*	*Std.Dev.*		*Estimate*	*Std.Error*		*Estimate*	*Std.Dev.*	
Country (random intercept)	0.176	0.420		0.123	0.351		0.103	0.321	
Wave	0.037	0.191		0.044	0.209		0.041	0.202	
Fixed effects	*Estimate*	*Std.Dev.*	*P-value*	*Estimate*	*Std.Dev.*	*P-value*	*Estimate*	*Std. Error*	*P-value*
(Intercept)	1.080	0.383	0.005	-0.743	0.383	0.053	0.092	0.377	0.808
Wave(1=2017)	0.560	0.112	0.000	0.431	0.119	0.000	0.420	0.116	0.000
Age	0.005	0.010	0.623	0.041	0.010	0.000	0.018	0.010	0.066
Sex(women=1)	0.216	0.085	0.011	0.229	0.083	0.006	0.154	0.081	0.058
Age squared	0.000	0.000	0.000	0.000	0.000	0.000	0.000	0.000	0.000
High education (ref. low)				1.129	0.129	0.000	1.196	0.127	0.000
Medium education (ref. low)				0.965	0.096	0.000	1.000	0.095	0.000
Ever smoked							0.091	0.010	0.000
BMI							-0.002	0.001	0.058
Depression (eurod)							-0.040	0.002	0.000
Physical inactivity							-0.316	0.013	0.000
Hearing (1-excellent, 5-poor)							-0.040	0.005	0.000
Wave:age	-0.004	0.001	0.003	-0.003	0.001	0.031	-0.003	0.001	0.021
Wave:sex	0.029	0.021	0.172	0.001	0.021	0.944	-0.001	0.020	0.970
Age:sex	-0.005	0.001	0.000	-0.004	0.001	0.001	-0.002	0.001	0.134
Age:high educ.				-0.007	0.002	0.000	-0.009	0.002	0.000
Age:medium educ				-0.009	0.001	0.000	-0.010	0.001	0.000
Wave:high educ.				-0.013	0.033	0.700	-0.006	0.032	0.844
Wave:medium educ.				0.027	0.026	0.297	0.037	0.026	0.151

Supplementary Table 4. Conditional quantile regression for outcomes: immediate recall, delayed recall, and verbal fluency. Covariates: education, wave (year/cohort), age and sex. n=32 773

	Quantile								
**Immediate recall**	10	20	30	40	50	60	70	80	90
Low education									
Intercept	2.259	2.471	2.314	2.272	2.483	2.694	2.667	3.201	3.539
Wave (1=2017)	0.274	0.287	0.274	0.253	0.253	0.211	0.235	0.211	0.211
Age	-0.055	-0.052	-0.046	-0.042	-0.042	-0.042	-0.039	-0.042	-0.042
Sex (1=women)	0.055	0.105	0.046	0.084	0.084	0.127	0.157	0.127	0.169
Medium education									
Intercept	2.298	2.131	2.205	2.121	2.607	2.508	2.734	3.172	3.515
Wave (1=2017)	0.274	0.224	0.183	0.194	0.146	0.161	0.129	0.103	0.069
Age	-0.048	-0.041	-0.037	-0.032	-0.037	-0.032	-0.032	-0.034	-0.034
Sex (1=women)	0.226	0.285	0.183	0.161	0.256	0.226	0.226	0.206	0.274
High education									
Intercept	2.001	2.040	2.153	2.520	2.760	2.863	3.089	2.945	3.384
Wave (1=2017)	0.274	0.240	0.258	0.240	0.206	0.233	0.161	0.220	0.183
Age	-0.039	-0.034	-0.032	-0.034	-0.034	-0.033	-0.032	-0.027	-0.028
Sex (1=women)	0.274	0.206	0.194	0.240	0.171	0.216	0.161	0.192	0.183
**Delayed recall**									
Low education									
Intercept	-1.621	0.897	1.898	2.586	2.615	2.762	2.728	3.071	3.435
Wave (1=2017)	0.000	0.091	0.121	0.162	0.177	0.200	0.175	0.243	0.243
Age	0.000	-0.030	-0.040	-0.046	-0.044	-0.043	-0.039	-0.040	-0.040
Sex (1=women)	0.000	0.061	0.121	0.092	0.132	0.128	0.097	0.121	0.162
Medium education									
Intercept	1.615	2.262	2.302	2.299	2.331	2.710	2.348	2.633	3.033
Wave (1=2017)	0.081	0.177	0.202	0.187	0.208	0.224	0.286	0.314	0.343
Age	-0.040	-0.044	-0.040	-0.037	-0.035	-0.037	-0.029	-0.029	-0.029
Sex (1=women)	0.243	0.265	0.243	0.261	0.243	0.336	0.257	0.228	0.200
High education									
Intercept	2.174	2.223	2.227	2.470	2.609	2.927	3.059	3.621	3.524
Wave (1=2017)	0.132	0.214	0.243	0.312	0.381	0.395	0.381	0.427	0.453
Age	-0.044	-0.039	-0.035	-0.035	-0.035	-0.036	-0.035	-0.039	-0.032
Sex (1=women)	0.309	0.252	0.243	0.173	0.277	0.252	0.347	0.291	0.324
**Verbal fluency**									
Low education									
Intercept	0.592	0.936	1.143	1.382	1.555	1.806	2.000	2.405	2.954
Wave (1=2017)	0.000	0.045	0.062	0.086	0.105	0.119	0.119	0.121	0.067
Age	-0.027	-0.028	-0.028	-0.029	-0.029	-0.030	-0.030	-0.032	-0.034
Sex (1=women)	-0.081	-0.062	-0.067	-0.077	-0.077	-0.075	-0.045	-0.026	-0.034
Medium education									
Intercept	0.832	1.344	1.776	2.015	2.283	2.533	2.887	3.381	4.430
Wave (1=2017)	0.034	0.027	0.060	0.095	0.142	0.173	0.168	0.220	0.224
Age	-0.025	-0.027	-0.030	-0.030	-0.032	-0.032	-0.034	-0.037	-0.045

Sex (1=women)	0.025	0.000	0.000	0.009	0.016	0.013	0.000	0.000	-0.045
High education									
Intercept	1.344	1.532	2.118	2.449	2.680	2.988	3.442	3.981	4.373
Wave (1=2017)	0.030	0.027	0.021	0.083	0.096	0.134	0.146	0.182	0.192
Age	-0.030	-0.027	-0.031	-0.032	-0.032	-0.034	-0.037	-0.039	-0.038
Sex (1=women)	0.045	0.134	0.134	0.089	0.077	0.101	0.098	0.055	0.077

Supplementary Table 5. Standard deviations in immediate recall (a), delayed recall (b), and verbal fluency (c) scores in 2007 and 2017. Calculated from non-standardised variables.

Delayed Recall

	Wave 2 (2007)	Wave 7 (2017)
60-64	1.65	1.58
65-69	1.67	1.53
70-74	1.67	1.60
75-79	1.72	1.66
80-84	1.79	1.74
85-89	1.80	1.81
90-94	1.76	1.88

Immediate recall

	Wave 2 (2007)	Wave 7 (2017)
60-64	1.90	2.05
65-69	1.90	1.97
70-74	1.82	2.04
75-79	1.83	1.99
80-84	1.80	1.99
85-89	1.66	1.90
90-94	1.48	1.72

Verbal fluency

	Wave 2 (2007)	Wave 7 (2017)
60-64	7.15	7.77
65-69	7.02	7.57
70-74	6.70	7.21
75-79	6.52	7.37
80-84	6.72	7.10
85-89	6.22	6.84
90-94	6.67	7.57

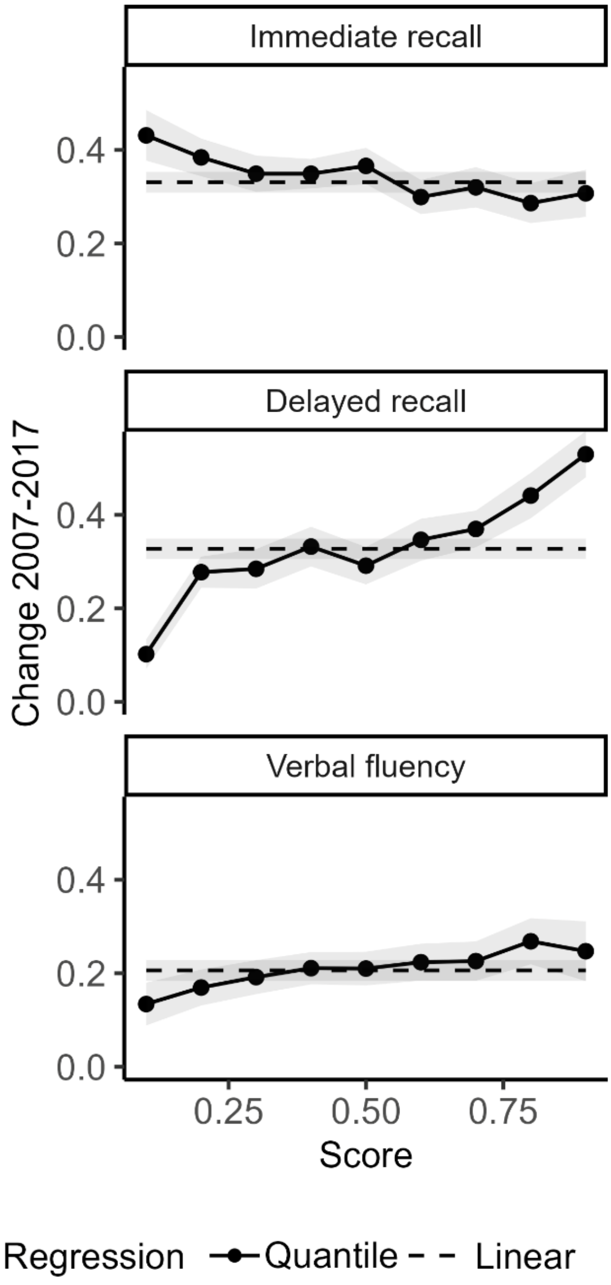

Supplementary Figure 6. Change in immediate recall delayed recall, , and verbal fluency between 2007 and 2017 in the entire sample. Dotted line shows the average change in the entire sample estimated from a linear regression, full solid lines show changes at every 10th percentile estimated from quantile regression. Grey bands indicate 95 % Confidence intervals. Adjusted for age and sex (n=32 773).
